# A study on neurotransmitter levels in rats with Tic disorder treated with aripiprazole

**DOI:** 10.3389/fnins.2025.1691717

**Published:** 2025-11-03

**Authors:** Hui-Min Chen, Yang Wang, Ying-Ying Xin, Dan Sun, Xue Yang, Zhi-Sheng Liu

**Affiliations:** ^1^Department of Hyperbaric Oxygen, Shenzhen Children's Hospital, Shenzhen, China; ^2^Department of Pharmacy, Wuhan Childrens Hospital of Tongji Medical College, Huazhong University of Science and Technology, Wuhan, China; ^3^Department of Child Healthcare, Wuhan Childrens Hospital of Tongji Medical College, Huazhong University of Science and Technology, Wuhan, China; ^4^Department of Neurology, Wuhan Childrens Hospital of Tongji Medical College, Huazhong University of Science and Technology, Wuhan, China

**Keywords:** aripiprazole, Tic disorders, neurotransmitter, children, rats

## Abstract

**Background:**

Tic disorder (TD) involves dysregulation of neurotransmitters. Although aripiprazole (ARI) is a first-line therapy, its mechanism remains debated, and animal studies are limited. This study investigates ARI's effects on neurotransmitter levels in a rat TD model.

**Methods:**

Forty male Sprague-Dawley rats were randomly assigned to five groups of eight animals each: control group, TD untreated group, and low/medium/high dose ARI groups (1.6/4/10 mg/kg). With the exception of the control group, all rats underwent modeling with 3,3'-iminodipropionitrile (IDPN) for seven consecutive days.Following modeling, the ARI groups were administered different doses of ARI via gavage for 2 consecutive weeks. The control group and TD untreated group received equivalent volumes of saline. Behavioral tests were conducted upon completion of the modeling phase and again following the 2-week gavage period. Changes in neurotransmitter levels in rat plasma and striatum were detected using ultra-performance liquid chromatography-tandem mass spectrometry (UPLC-MS/MS).Receiver operating characteristic (ROC) curve analysis was performed on neurotransmitters showing statistically significant differences to evaluate their diagnostic efficacy for TD.

**Results:**

(1) Compared with the TD untreated group, all ARI dose groups showed lower motor and stereotypic behavior scores after the 14-day gavage period. (2) As shown by UPLC-MS/MS, the TD untreated group had significantly lower glutamate (Glu) and γ-aminobutyric acid (GABA) levels in plasma, yet higher levels in the striatum, when compared to the control group. (3) Following ARI intervention, a marked reduction in striatal Glu and GABA levels was observed across all dose groups, and the Glu/GABA ratio showed a dose-dependent reduction. (4) ROC analysis revealed that plasma Glu and GABA alone had moderate predictive efficacy; striatal Glu and GABA demonstrated superior predictive performance. When combined for prediction, the AUC for plasma neurotransmitters was 0.848, and for striatal neurotransmitters, it was 0.938.

**Conclusion:**

ARI can regulate motor and stereotypic behaviors in TD rats and effectively control tic symptoms. TD rats exhibit dysregulation of Glu and GABA levels, and ARI can modulate Glu and GABA levels, thereby improving neurotransmitter function. Furthermore, central neurotransmitter changes demonstrate superior diagnostic value over peripheral measures for TD.

## 1 Introduction

Tic disorders (TD) are neurodevelopmental disorders. Based on distinct clinical characteristics and duration, TD can be classified into three primary types: provisional TD, chronic TD, and Tourette syndrome (TS) (Liu et al., 2020). Among these, TS is the most severe form, with a prevalence rate of 0.4% (Li et al., 2022). The underlying pathophysiological mechanisms are closely associated with dysfunction in the cortico-striato-thalamo-cortical (CSTC) circuit and imbalances in neurotransmitter systems (Rizwan et al., 2022). These neurotransmitters serve as biomarkers for assessing disease state and treatment efficacy (De Vries et al., 2022; Patel et al., 2022). The atypical antipsychotic aripiprazole (ARI) exerts its effects through a unique mechanism of action, functioning as a partial agonist at both dopamine D2 and 5-HT1A receptors (Zhang et al., 2024). The US Food and Drug Administration (FDA) granted approval for its use in treating TS in 2014 (Cavanna, 2022). European guidelines (Roessner et al., 2022), supported by moderate-quality evidence, recommend ARI as first-line therapy for pediatric and adult TD due to its established efficacy in reducing severity and favorable side effect profile.

However, the precise neurobiological mechanisms underlying ARI therapeutic effects in TD remain incompletely understood. Currently, there is a scarcity of research, both domestically and internationally, investigating changes in neurotransmitter levels in TD rat models following ARI treatment. Moreover, animal studies rarely incorporate dynamic monitoring of neurotransmitter levels during the modeling phase itself. Additionally, obtaining cerebrospinal fluid from pediatric TD patients presents significant clinical challenges. Due to factors such as the blood-brain barrier, differences in neurotransmitter metabolism, and disparate tissue sources, clinical studies relying solely on blood samples for neurotransmitter analysis may not accurately reflect central nervous system alterations (Braga et al., 2024). Thus, this study establishes a validated TD rat model simulating human TD pathophysiological features. We measure neurotransmitter levels in both plasma and brain tissue before and after ARI treatment, investigating the potential mechanism of ARI-mediated symptom improvement through neurotransmitter modulation. This research aims to provide laboratory evidence for further elucidating TD pathogenesis and the therapeutic mechanisms of ARI.

## 2 Materials and methods

### 2.1 Experimental animals

Forty male Sprague-Dawley(SD) rats (21 days old, initial body weight (60 ± 10) g) were provided by Hubei Bennte Biological Technology Co., Ltd. (Animal Production License No.: SCXK(E)2021-0027). The facility usage license number was SYXK(E)2021-0057. Animals were housed under standardized conditions in a specific pathogen-free (SPF) barrier system (room temperature 20–25 °C, humidity 50%, 12 h light and dark period), free feeding and drinking water, and the experiment was carried out after 5 days of adaptive feeding. This study was conducted following the guidelines approved by the Animal Experiment Ethics Committee of Huazhong University of Science and Technology (Ethics No. 4058) for all animal experiments.

### 2.2 Main instruments and reagents

Low-temperature high-speed refrigerated centrifuge (Beijing Baiyang Medical Equipment Co., Ltd.); Capillary sampling tubes (Zhejiang Gongdong Medical Instrument Co., Ltd.); Gavage needles (Beijing Vitone Lihua Laboratory Animal Technology Co., Ltd.); Aripiprazole Oral Solution (Sichuan Otsuka Pharmaceutical Co., Ltd., 150 ml/bottle); 3,3′-Iminodipropionitrile (IDPN, Sigma-Aldrich, USA).

### 2.3 Research methods

#### 2.3.1 Animal grouping and drug intervention

Preparation of IDPN involved dissolution in saline to yield a 25 mg/ml stock solution. Daily intraperitoneal administration of this solution (250 mg/kg) was performed for 7 days.The control group received equivalent volumes of saline concurrently, once daily for 7 days. Twenty-four hours after the last injection, the modeling group survival rate was 100%, with no significant weight fluctuation (Δ BW < 5%) or feeding inhibition. All modeled rats exhibited characteristic movements and stereotypic movements, with behavioral scores≥2 points, meeting internationally recognized criteria for successful TD animal modeling.

**Figure 1:**
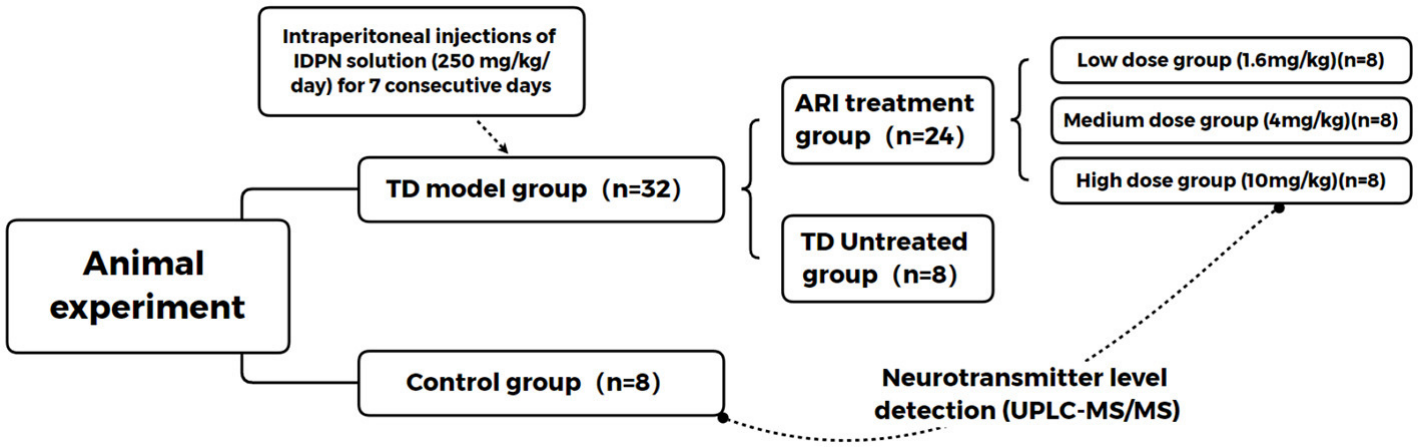
Animal grouping and experimental design.Schematic representation of the experimental groups and treatment timeline.Forty male SD rats were randomly assigned to five groups (*n* = 8 per group): control group, TD untreated group, and three ARI treatment groups (low dose: 1.6 mg/kg, medium dose: 4 mg/kg, high dose: 10 mg/kg). TD model was established by intraperitoneal injection of IDPN (250 mg/kg/day) for 7 consecutive days. ARI treatment was administered via gavage for 14 days after successful modeling. Neurotransmitter levels were detected using UPLC-MS/MS.

We randomly assigned the 32 TD model rats to four groups: an untreated group and three groups receiving ARI at doses of 1.6 (low), 4 (medium), and 10 (high) mg/kg. Eight non-modeled SD rats served as the control group (Figure 1). Starting from experimental day 8, the low, medium, and high dose ARI treatment groups were administered different concentrations of ARI via gavage. The drug was diluted with saline to a gavage volume of 5 ml/kg. The control group and untreated group received equivalent volumes of saline via gavage, once daily for 2 consecutive weeks.

#### 2.3.2 Body weight

Rat body weight changes (g) were measured and recorded at the end of the 7-day IDPN modeling period, and at 7 days and 14 days after starting ARI gavage.

#### 2.3.3 Behavioral scoring

Referring to the behavioral evaluation criteria for TD animal models reported by Diamond et al. (1982), Quiet, dark environment, behavioral scoring was carried out on 7 days of modeling and 14 days of ARI gavage, and each rat was observed to adapt to the environment in the cage for 5 min and then recorded the activities of the rats in any 1 h with camera equipment, recorded every 5 min, and the activity was recorded for 5 min each time. After the test, the excrement in the cage was cleaned to eliminate the influence of the next rat, and the whole process was recorded by double-blind observation, and the data was observed separately by two people to reduce subjective error. Specific scoring criteria were as follows:

(1) Motor Behavior Score:

0 points: Quiet or normal activity;
1 point: Hyperactivity;
2 points: Increased exploratory behavior;
3 points: Running;
4 points: Running and jumping.

(2) Stereotypic Behavior Score:

0 points: No stereotypic behavior;
1 point: Rotational behavior;
2 points: Pronounced vertical excursion of the head and neck;
3 points: Pronounced vertical excursion of the head and neck combined with rotational behavior;
4 points: Head tilting to the side and pronounced vertical excursion of the head and neck.

#### 2.3.4 Determination of ARI concentration and neurotransmitters in rat plasma and striatum

Orbital blood sampling was performed on rats from each group on Day 0 and at Week 1, prior to blood collection, the rats were anesthetized by intraperitoneal injection of 20% urethane solution at a dose of 10 mL/kg. EDTA-anticoagulated tubes containing rat orbital blood were centrifuged (3,000 rpm, 10 min, 4 °C). Collection a nd storage of 800 μL of supernatant were performed at –80 °C after centrifugation. At the end of Week 2, the rats in each group were anesthetized again (using the same procedure as above) prior to orbital blood collection, followed immediately by euthanasia. Brains were rapidly removed after decapitation, and bilateral striata were quickly dissected on a freezing stage, weighed, and homogenized in five volumes of HBSS buffer. After centrifugation (12,000 rpm for 10 min at 4 °C), 800 μL of the resulting supernatant was aliquoted for storage at –80 °C. All samples were transported under low temperature to Wuhan Kangshengda Biotechnology Co., Ltd. for detection of plasma and striatal ARI concentrations, as well as neurotransmitter levels.

#### 2.3.5 Statistical analysis

We employed the Shapiro–Wilk test to determine if continuous variables were normally distributed. Body weight and motor scores were normally distributed, while neurotransmitter levels were non-normally distributed. Continuous variables that followed a normal distribution were reported as mean ± standard deviation.We performed one-way ANOVA for multi-group comparisons, applying Bonferroni *post-hoc* tests for significant results. The Mann–Whitney *U* test and the Kruskal–Wallis test were employed for two-group and multi-group comparisons of non-normally distributed data, expressed as median (interquartile range) [M (P25, P75)]. When the omnibus Kruskal–Wallis test was significant, Bonferroni-adjusted pairwise comparisons were conducted. Receiver operating characteristic (ROC) curves were plotted for neurotransmitter levels showing statistically significant differences, and the area under the curve (AUC) was calculated as an evaluation index to assess the diagnostic efficacy of individual and combined neurotransmitters for TD. All statistical analyses and graph plotting were performed using SPSS 26.0 and Origin 2022. Statistical significance for all two-sided analyses was set at *P* < 0.05.

## 3 Results

### 3.1 Comparison of rat body weight

**Figure 2:**
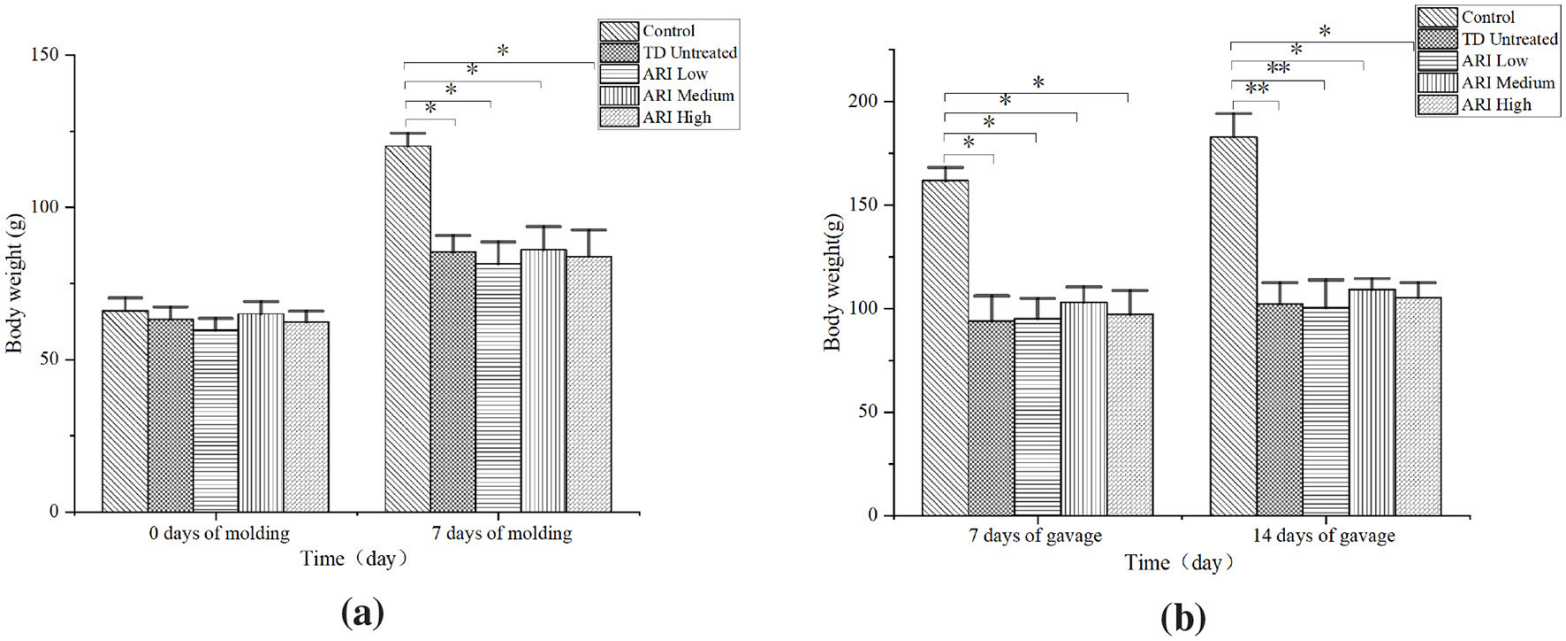
Body weight changes during modeling and gavage periods. **(a)** Comparison of body weight at 0 and 7 days of IDPN modeling (*n* = 8 per group). **(b)** Comparison of body weight after 7 and 14 days of ARI gavage (*n* = 8 per group). Body weight was measured using electronic scales. Data are presented as mean ± SD. ^*^P < 0.05, ^**^P < 0.01 vs. control group.

On the 0th day of modeling, We found that body weight was comparable across the control, TD untreated, and all ARI dose groups. (*P* > 0.05). On the 7th day of modeling, 7 days of gavage and 14 days of gavage, the body weight of the rats in the TD untreated group and all ARI dose groups was consistently lower than that of the control group (*P* < 0.05) (Figure 2). At the same time, we observed no significant difference in rat body weight among the TD untreated and the various ARI dose groups. (*P* > 0.05).

### 3.2 Comparison of rat motor and stereotypic behavior scores

**Figure 3:**
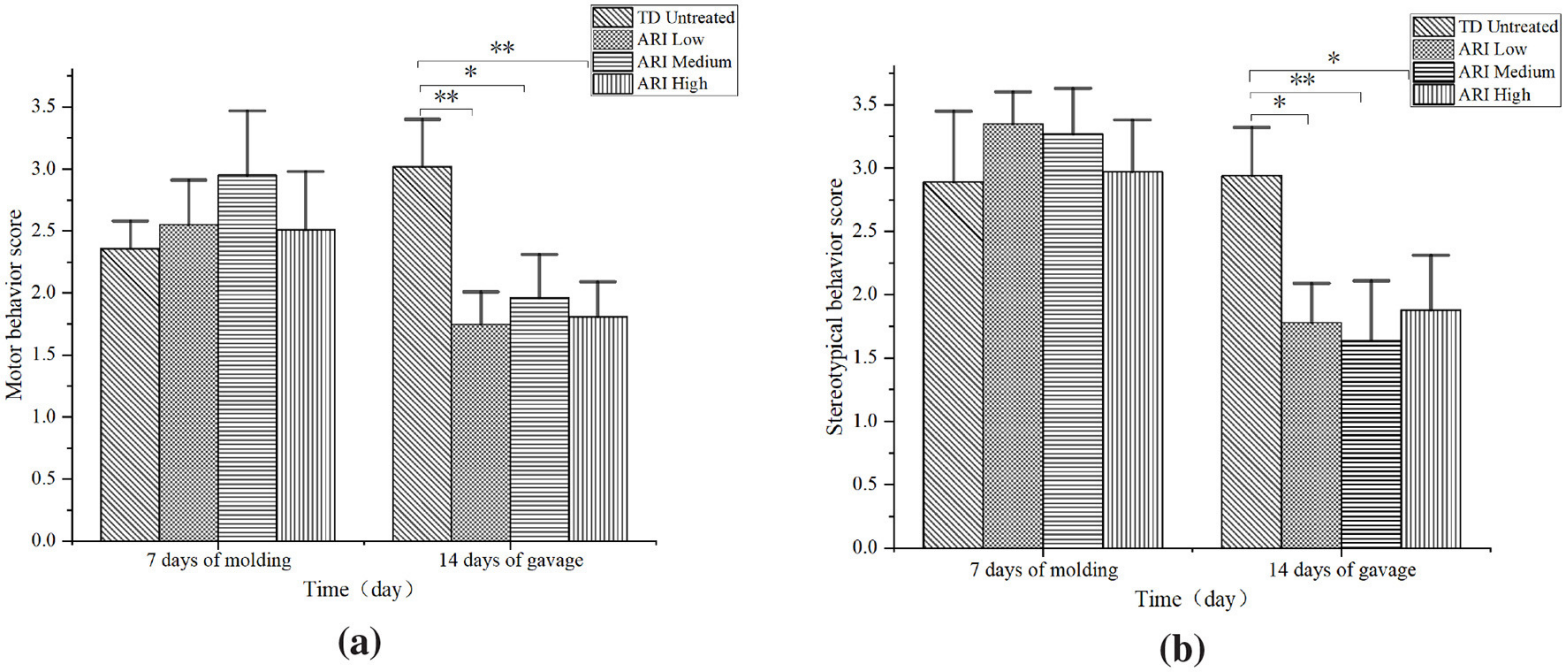
Motor and stereotypic behavior scores across groups. **(a)** Motor behavior scores during 7-day modeling and 14-day gavage periods (*n* = 8 per group). **(b)** Stereotypic behavior scores during the same periods. Behavioral assessment was performed using the Diamond scoring system in quiet, dark environment with double-blind observation. Data are presented as mean ± SD. ^*^P < 0.05, ^**^P < 0.01 vs. TD untreated group.

After 7 days of modeling, motor and stereotypic behavior scores showed no significant differences among the TD untreated group and the various ARI dose groups.(*P* > 0.05). After 14 days of gavage, all ARI dose groups exhibited significantly reduced motor and stereotypical behavior scores compared to the TD untreated group.(*P* < 0.05) (Figure 3). Meanwhile, we observed no significant difference in motor and stereotypic behavior scores among the various ARI dose groups. (*P* > 0.05).

### 3.3 Comparison of neurotransmitter levels among groups

**Figure 4:**
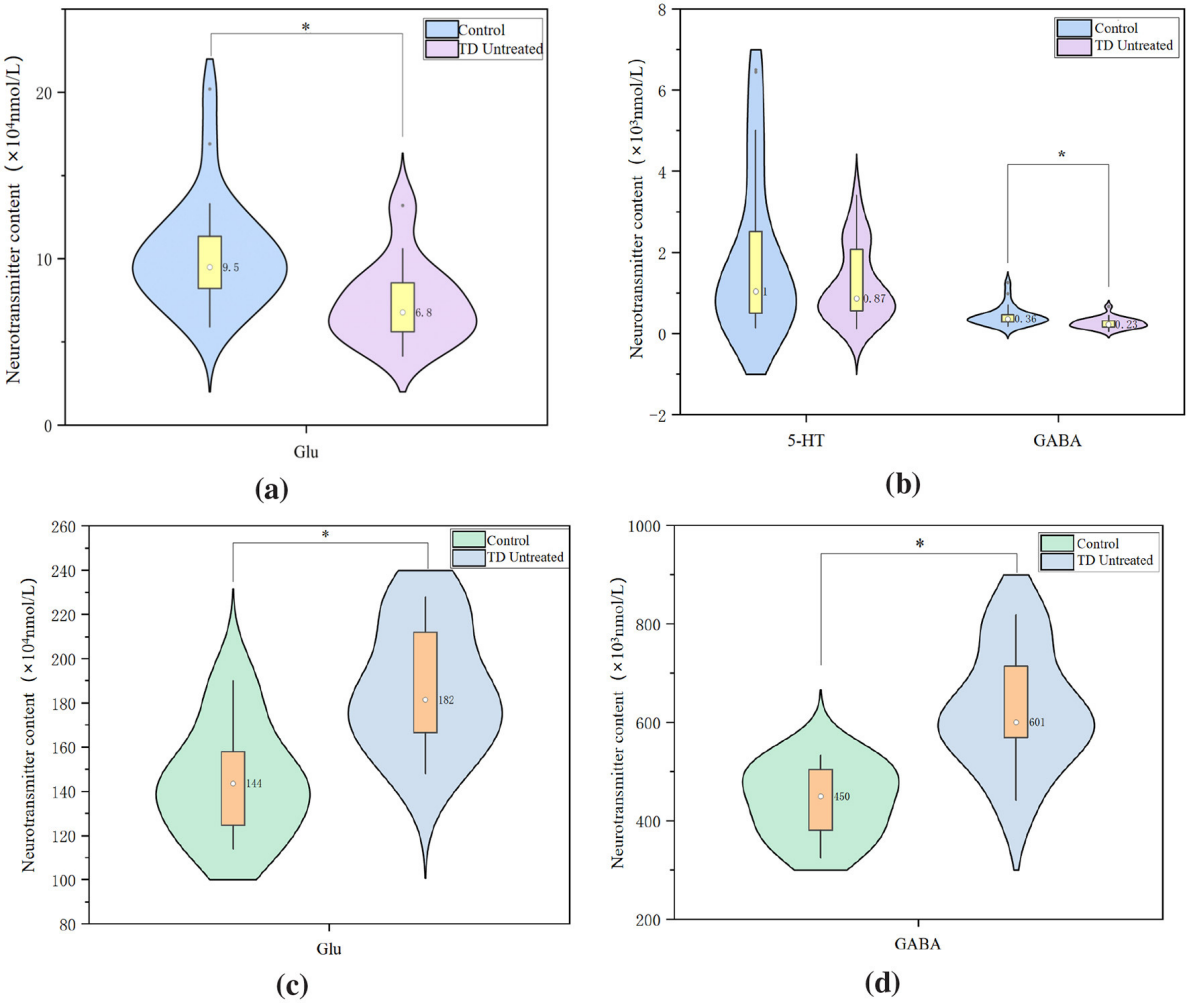
Neurotransmitter levels in plasma and striatum compared to control group.UPLC-MS/MS analysis of neurotransmitter concentrations (*n* = 8 per group). **(a)** Plasma Glu content. **(b)** Plasma GABA content. **(c)** Striatum Glu content. **(d)** Striatum GABA content. Data are presented as median with interquartile range. ^*^P < 0.05 vs. control group.

Compared to the control group (plasma Glu median: 9.49 × 10^4^ mmol/L, GABA median: 0.36 × 10^3^mmol/L), plasma Glu and GABA levels in the TD untreated group were significantly decreased to 6.78 × 10^4^mmol/L (*P* = 0.027) and 0.23 × 10^3^ mmol/L (*P* = 0.039), respectively. Compared to the control group (striatal Glu: 143.50 × 10^4^ mmol/L, GABA: 450.00 × 10^3^ mmol/L), striatal Glu and GABA levels in the TD untreated group were significantly increased to 181.50 × 10^4^mmol/L (*P* = 0.020) and 600.50 × 10^3^mmol/L (*P* = 0.016), respectively (Figure 4). The concentrations of dopamine (DA), epinephrine (E), norepinephrine (NE), and serotonin (5-HT) in both plasma and striatal tissue showed no significant changes.

**Figure 5:**
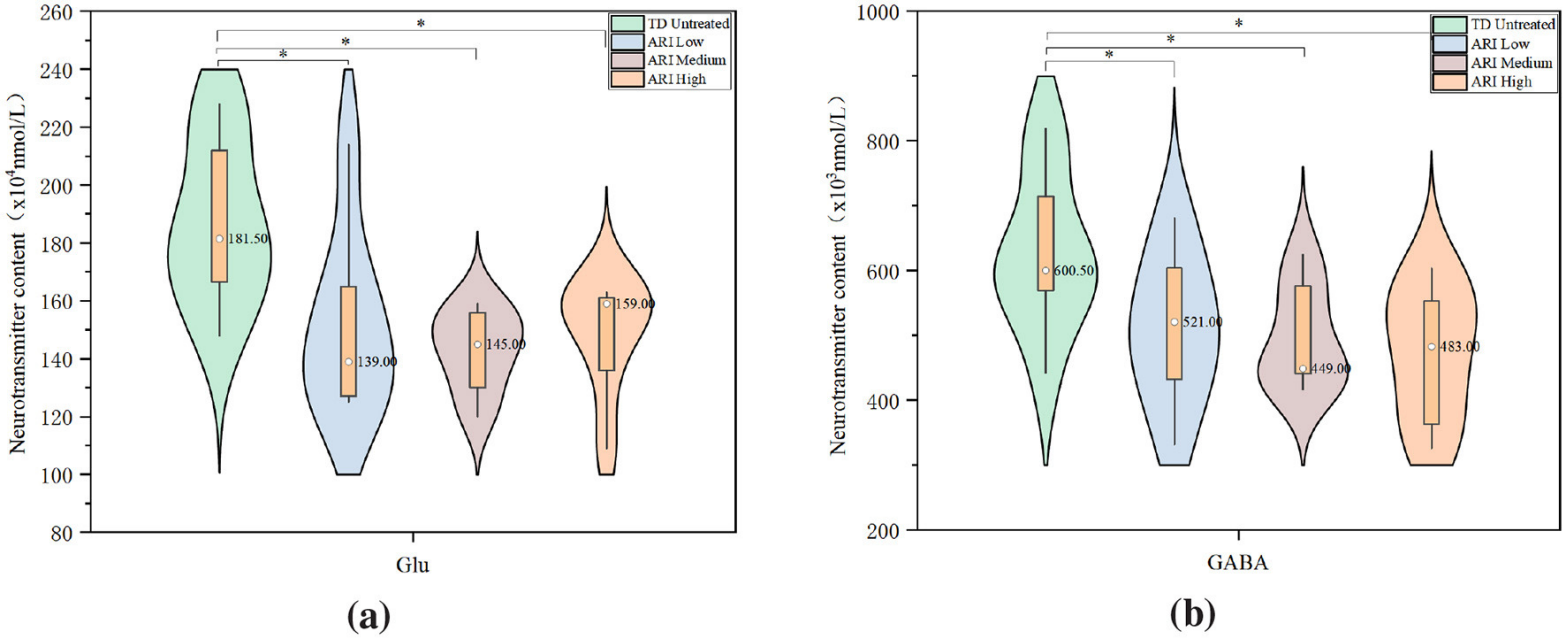
Effects of ARI treatment on striatal Glu and GABA levels.UPLC-MS/MS analysis of striatal neurotransmitter concentrations after 14 days of ARI treatment (*n* = 8 per group). **(a)** Glu levels across treatment groups. **(b)** GABA levels across treatment groups. Data are presented as median with interquartile range. ^*^p < 0.05 vs. TD untreated group.

Compared to the TD untreated group, there were no statistically significant differences in plasma DA, E, NE, 5-HT, Glu, and GABA levels among the low, medium, high dose ARI groups at 1 week and 2 weeks post-treatment. At 2 weeks post-treatment, striatal Glu levels in the low dose ARI group (139.00 × 10^4^mmol/L, *P* = 0.010), medium dose group (145.00 × 10^4^ mmol/L, *P* = 0.014), and high dose group (159.00 × 10^4^mmol/L, *P* = 0.019) were all lower than in the TD untreated group (181.50 × 10^4^ mmol/L). Striatal GABA levels in the low dose ARI group (521.00 × 10^3^ mmol/L, *P* = 0.027), medium dose group (449.00 × 10^3^ mmol/L, *P* = 0.012), and high dose group (490.00 × 10^3^ mmol/L, *P* = 0.021) were all lower than in the TD untreated group (600.50 × 10^3^ mmol/L) (Figure 5) . There were no statistically significant differences in striatal DA, E, NE, and 5-HT levels among the groups.

**Figure 6:**
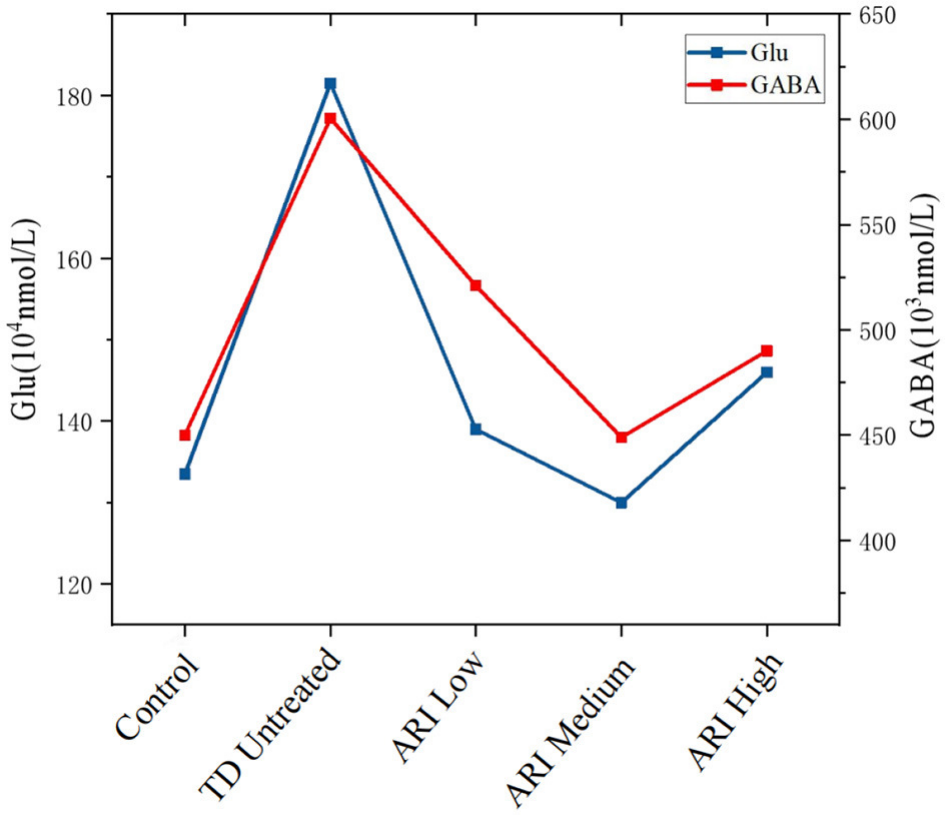
Line chart showing levels of Glu (glutamate) and GABA across five conditions: Control, TD Untreated, ARI Low, ARI Medium, and ARI High. Glu, represented by a blue line, peaks at TD Untreated and declines through ARI High. GABA, shown by a red line, also peaks at TD Untreated and follows a similar decline, with a slight rise from ARI Medium to ARI High. Glu levels range from 140 to 180 (10^4^ nmol/L) and GABA levels from 400 to 650 (10^3^ nmol/L). Striatal Glu and GABA levels across groups.Line chart showing striatal neurotransmitter concentrations in different treatment groups (*n* = 8 per group). UPLC-MS/MS analysis was performed on striatal tissue homogenates. Data represent median values from 8 animals per group.

The concentrations of Glu and GABA in the striatum of rats in each group are shown in Figure 6, and the proportion of Glu/GABA in the low-dose group (26.68%), medium-dose group (28.95%) and high-dose group (29.79%) decreased compared with the TD untreated group (30.22%), but there was no statistical difference (*P* > 0.05).

### 3.4 Diagnostic efficacy evaluation of neurotransmitters

ROC curve analysis was performed on neurotransmitters that showed statistically significant differences between the TD untreated group and the control group in plasma and striatum (Glu and GABA) to evaluate their diagnostic efficacy for TD. The results showed:

For plasma neurotransmitters, the AUCs for Glu and GABA were 0.793 (95% CI: 0.662, 0.925) and 0.777 (95% CI: 0.645, 0.909), with sensitivities of 87.0% and 78.3%, and specificities of 66.7% and 70.8%, respectively, indicating moderate predictive performance. For striatal neurotransmitters, the AUCs for Glu and GABA were 0.891 (95% CI: 0.721, 1.000) and 0.938 (95% CI: 0.808, 1.000), with sensitivities of 87.5% and 87.5%, and specificities of 75% and 87.5%, respectively, indicating good predictive performance.

**Figure 7:**
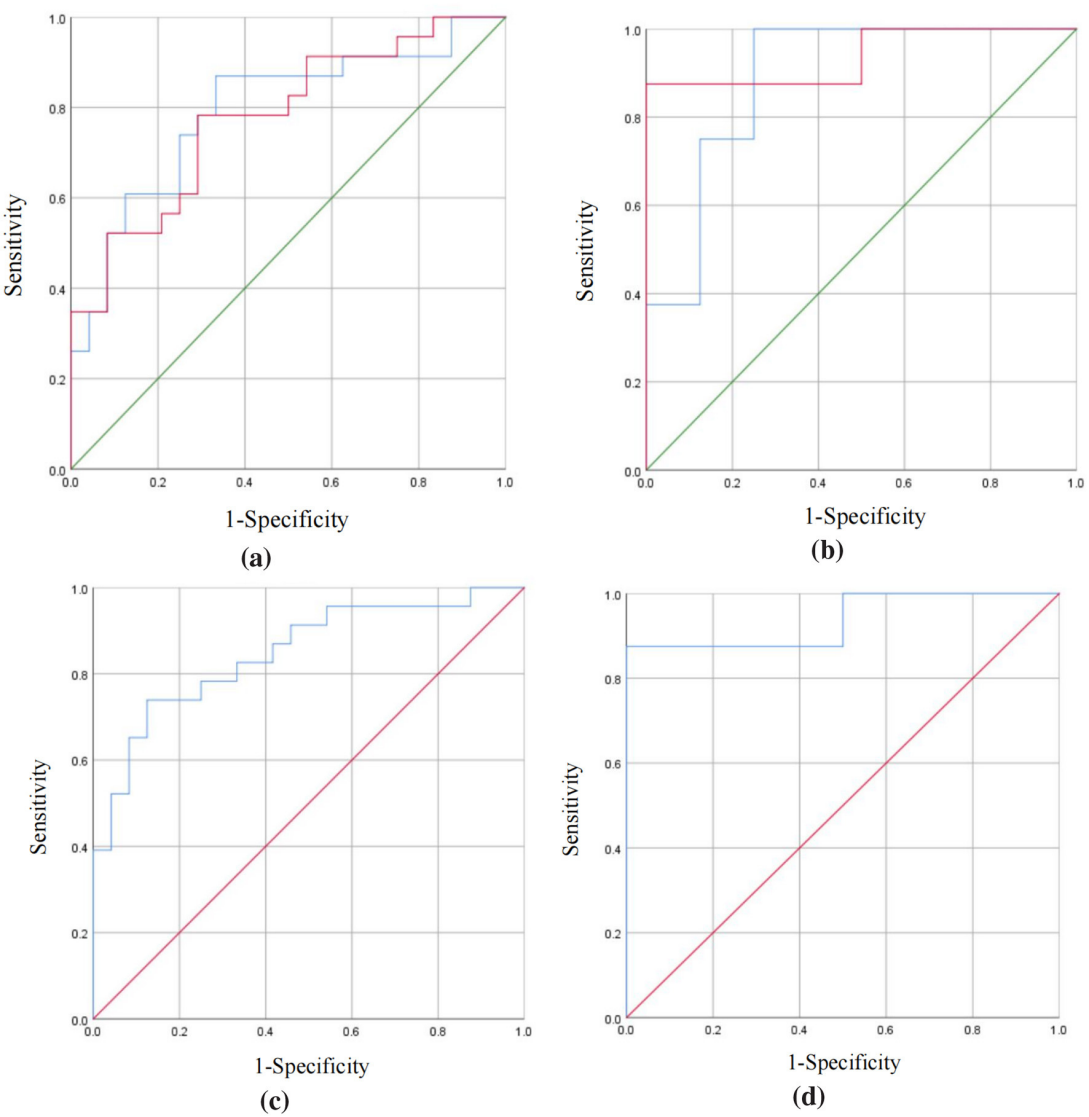
Four ROC curves are shown, labeled (a) through (d). Each plot has sensitivity on the Y-axis and 1-specificity on the X-axis. Diagonal green lines represent random classifiers, while blue and red lines depict the performance of different models. Panels (a) and (b) show more variations in curves, indicating model performance differences. Panels (c) and (d) have fewer fluctuations, suggesting a stronger classifier consistency. ROC curve analysis of neurotransmitter diagnostic efficacy for TD. **(a)** Plasma neurotransmitters: Glu (AUC = 0.793) and GABA (AUC = 0.777). The blue line represents Glu, the red line represents GABA, and the green line is the reference line. **(b)** Striatal neurotransmitters: Glu (AUC = 0.891) and GABA (AUC = 0.938). Line colors correspond to those in **(a)**. **(c)** Combined plasma neurotransmitters (AUC = 0.848). The blue line represents the combination marker (Glu and GABA), and the red line is the reference line. **(d)** Combined striatal neurotransmitters (AUC = 0.938). Line colors correspond to those in **(c)**.

The integration of plasma neurotransmitters resulted in an AUC of 0.848 (95% CI: 0.736, 0.960), demonstrating 73.9% sensitivity and 87.5% specificity. In contrast, the combination of striatal neurotransmitters achieved a higher AUC of 0.938 (95% CI: 0.808, 1.000), with 87.5% sensitivity and 75.0% specificity (Figure 7).

## 4 Discussion

### 4.1 TD rat model dosing and methodology

Currently established methods for constructing TD models include both induced and spontaneous animal models, such as those induced by neurotransmitter abnormalities or immune dysregulation, as well as genetically engineered spontaneous models (Xu et al., 2022). However, none fully recapitulate all manifestations of TD (Kowalski et al., 2025). Among these, the IDPN-induced model exhibits behavioral phenotypes highly consistent with clinical TD children. Established by Diamond et al. in 1982, this model is considered ideal due to its simplicity, long-lasting effects, high reproducibility, and irreversible nature. Its behavioral manifestations align with the clinical onset and progression of TD, including hyperactivity, stereotypies, and difficulty adapting to environments, demonstrating significant, stable, and persistent face validity (Yu et al., 2023). Previous literature (Yang et al., 2022; Wu et al., 2025; Wang et al., 2023) reported common IDPN doses ranging from 150-350 mg/kg, with higher doses correlating with increased mortality risk in rats. The most prevalent modeling duration was 7 days, primarily using SD rats. Three dose gradients (150, 250, and 350 mg/kg) were tested in our preliminary experiments. Rats receiving 150 mg/kg IDPN showed minimal tic-like symptoms, while those receiving 350 mg/kg exhibited significant reductions in food intake and body weight. Conversely, administration of 250 mg/kg IDPN for 7 days successfully induced tic-like behavioral manifestations in rats, such as head jerks and rotations, without adverse effects like reduced feeding or weight loss.

### 4.2 Efficacy of ARI treatment in TD rats

This study demonstrates that ARI exhibits significant therapeutic efficacy in the TD rat model. Notably, after 14 days of intragastric administration, ARI showed pronounced improvements in both locomotor activity and stereotyped behaviors. For clinicians, it is important to note that direct mg–mg comparisons between species are pharmacologically ineffective due to differences in metabolism, bioavailability, and body surface area. The ARI doses used in this study (1.6, 4, and 10 mg/kg) were carefully selected based on the principle of allometric amplification in pharmacokinetics, as well as previous pre-experiments that explain these interspecific differences. According to FDA dose conversion guidelines, animal doses can be converted to human equivalents using species-specific conversion factors. Furthermore, the impact of ARI on these behavioral indices did not differ substantially across different doses, indicating favorable dose stability. However, the current experiment only observed the therapeutic effects over 14 days. Future studies should include longer-term follow-up to assess the sustained efficacy and safety profile of ARI. Additionally, further exploration is warranted to determine the optimal therapeutic dose range of ARI and to investigate the specific receptors or signaling pathways through which ARI ameliorates behavioral abnormalities in TD rats. These findings support the clinical application of ARI for TD treatment.

### 4.3 Changes in neurotransmitter levels in TD rats post-ARI treatment

Previous studies have validated the use of LC-MS/MS for quantifying rat neurotransmitters, highlighting its advantages of high sensitivity, specificity, precision, accuracy, and broad applicability. For instance, Zhai et al. (2015) developed an LC-MS/MS method to simultaneously quantify 11 deficient neurotransmitters in the urine of chronically stressed rats, finding significantly elevated tryptophan and tyrosine concentrations compared to controls. Fu et al. (2021) developed and validated a UPLC-MS/MS method combined with *in vivo* microdialysis to simultaneously determine lignans from Schisandra Chinensis and their effects on neurotransmitter levels in Alzheimer's disease rats. Xhakaza et al. (2021) also achieved successful quantification of buprenorphine and neurotransmitter levels in rat brain using UPLC-MS/MS. In this study, we similarly employed LC-MS/MS to measure neurotransmitter levels in the brains of TD rats following ARI treatment.

The findings showed that relative to the control group, the TD untreated group showed decreased serum Glu and GABA levels but increased striatal Glu and GABA. After 2 weeks of treatment, ARI-treated TD rats (low, medium, and high dose groups) showed reduced striatal Glu and GABA levels compared to the TD untreated group, although these differences were not statistically significant between the ARI dose groups. Previous studies have reported that traditional Chinese medicines like Jianpi Zhidong Granules, Gastrodia elata, and Jing'an Oral Liquid also reduce striatal Glu and GABA levels in TD rats post-treatment, potentially by influencing receptor expression and related pathways. Wen et al. (2016) suggested Jianpi Zhidong Granules maintain Glu and GABA levels by modulating their receptor expression in TD rats. Long et al. (2019) found Gastrodia elata significantly lowered stereotypy scores and reduced Glu and GABA content, possibly by regulating changes in striatal Nrf-2/HO-1/HMGB1/NF-κB pathway proteins in TS rats. Xi et al. (2022) demonstrated that Jing'an Oral Liquid significantly decreased Glu and GABA levels in TS rats, potentially linked to inhibiting microglial activation and modulating the NMDAR/MAPK/CREB protein pathway. Our findings align with these studies, showing reduced striatal Glu and GABA after ARI treatment. However, some studies report differing results; for example, Qinglong Zhidong Decoction was found to ameliorate tic-like behaviors in mice by restoring gut microbiota and modulating neurotransmitter balance, significantly reducing excitatory neurotransmitters Glu and DA while increasing inhibitory GABA (Wang et al., 2022). These inconsistencies may arise because Glu and GABA levels are influenced by multidimensional regulatory mechanisms. These include molecular mechanisms like abnormalities in neurotransmitter biosynthesis pathways and synaptic homeostasis dysregulation, as well as interactions with clinical disease stage, symptom spectrum characteristics, and treatment interventions. Furthermore, neurotransmitters exhibit region-specific distribution in the brain, and synaptic transmission is highly dynamic in space and time. Consequently, single time-point measurements in TD models reflect transient states, and their pathophysiological significance requires comprehensive evaluation with dynamic monitoring data.

Glu is the most important excitatory neurotransmitter in the central nervous system, which promotes neuronal excitation by activating NMDA receptors, etc., while GABA, as an inhibitory neurotransmitter, inhibits neuronal activity by activating GABAA receptors and GABAB receptors (Sheibani et al., 2022). Previous literature suggests reduced NMDAR levels in the cortex and striatum of TS rats (Caffino et al., 2025). The consistent directional change in excitatory Glu and inhibitory GABA observed in this study might be explained as follows: ARI possesses unique partial agonist activity at DRD2 and 5-HT1A receptors. It may prevent NMDAR antagonist-induced Glu release by activating the cystine/glutamate antiporter (Sxc)/Group II metabotropic glutamate receptor (II-mGluR) complex in the brain, ultimately leading to decreased Glu content (Fukuyama et al., 2018). Additionally, ARI can activate the mitogen-activated protein kinase (MAPK) and arachidonic acid signaling pathways (Stelmach et al., 2023). These pathways are implicated in GABA receptor activity and expression. Moreover, GABA is synthesized from glutamate via glutamate decarboxylase. Both mechanisms could contribute to the observed reduction in GABA content. This homotropic change pattern suggests that in the pathological state of TD, the relationship between Glu and the GABA system may not be a simple ”excitation-inhibition” antagonistic relationship. Instead, there are more complex synergistic or co-regulatory mechanisms.Our findings regarding ARI's modulation of striatal Glu and GABA levels should be considered in the context of its broader pharmacological profile. Emerging evidence indicates that ARI can inhibit inflammatory cytokine activity and modulate the nuclear factor-kappa-B/kynurenine pathway, thereby ameliorating inflammatory-mediated tissue damage and associated behavioral abnormalities (Mohammadgholi-Beiki et al., 2024). The neuroprotective properties of ARI, including its ability to reduce oxidative stress markers (malondialdehyde and myeloperoxidase), while enhancing glutathione levels and promoting the expression of neurotrophic factors (brain-derived neurotrophic factor, Nrf2, and Akt) (Mohammadgholi-Beiki et al., 2025), may contribute to its therapeutic efficacy in TD. Although our current study focused on neurotransmitter dynamics, these additional mechanisms could potentially complement ARI's effects on the CSTC circuit dysfunction in TD and warrant further investigation.

Furthermore, an elevated excitatory-to-inhibitory amino acid ratio is considered fundamental in the pathogenesis of neurodevelopmental disorders like TS, ASD, and OCD (Aida et al., 2015; Maier et al., 2022; Dou et al., 2021). Consequently, an imbalance in the Glu/GABA ratio within the CSTC circuit can lead to tic behaviors. This study found that compared to the TD untreated group, ARI-treated rats (all dose groups) exhibited a reduced striatal Glu/GABA ratio, further confirming that ARI can correct this imbalance. By modulating these neurotransmitter levels, ARI improves symptoms in tic rats. This finding provides new clues and insights for understanding the neurobiological mechanisms of TD and exploring more effective treatments.

### 4.4 Analysis of neurotransmitter differences in plasma vs. brain tissue and diagnostic efficacy evaluation

Neurotransmitters undergo distinct metabolic pathways and rates in plasma vs. brain tissue. Plasma neurotransmitters may originate from the peripheral nervous system or other tissues, whereas those in brain tissue directly reflect central nervous system activity. ARI has a plasma protein binding rate of approximately 99% and is rapidly absorbed, readily crossing the blood-brain barrier. Previous studies (Liang et al., 2012) found that after administering 10 mg/kg and 30 mg/kg ARI via gavage to healthy rats, the brain tissue concentration was approximately 5 times higher than the plasma concentration. This study also observed significant differences in ARI concentration between plasma and striatum in TD rats, and inconsistent changes in plasma vs. striatal neurotransmitters after ARI treatment. This further underscores that plasma neurotransmitter levels cannot substitute for brain tissue measurements in neurodevelopmental disorders, changes in brain tissue neurotransmitters are more representative. Therefore, brain tissue detection should be prioritized when assessing neurotransmitter changes.

In ROC curve analysis, a higher AUC value indicates better performance of the biomarker in distinguishing disease or state. This study found that the predictive efficacy of striatal (central) neurotransmitters, particularly GABA, was significantly higher than that of plasma (peripheral) neurotransmitters. This suggests that central neurotransmitter concentrations more directly reflect the core pathological mechanisms of the disease, while plasma neurotransmitters, potentially confounded by peripheral metabolism or blood-brain barrier limitations, serve only as indirect markers. Both Glu (AUC = 0.891) and GABA (AUC = 0.938) exhibited high predictive power (AUC > 0.8), with GABA's AUC approaching 0.94. This indicates GABA may play a “core regulatory molecule” role in the disease, where its concentration changes could directly correlate with tic severity or treatment response, warranting further validation of its potential as a therapeutic target. Combining plasma Glu and GABA enhanced predictive performance, suggesting that multi-neurotransmitter panels in the periphery might improve diagnostic accuracy through multi-pathway coverage. Conversely, combining central neurotransmitters did not enhance efficacy, possibly because a single central neurotransmitter (e.g., GABA) already provides sufficient sensitivity. In conclusion, Glu and GABA, as crucial neurotransmitters, exhibit varying predictive performance in different tissues. Future research could explore their application potential in diagnosing specific diseases, potentially integrating metabolomics or proteomics to screen for additional potential biomarkers and optimize combined predictive models for improved diagnostic accuracy.

### 4.5 Innovation and limitations

The innovative contribution of this study lies in its identification of striatal neurotransmitter (Glu and GABA) dysregulation as a key pathological feature in TD model rats and its demonstration that ARI ameliorates this dysfunction through modulation of Glu and GABA levels, thereby providing a potential research direction for developing neurotransmitter-based auxiliary diagnostic biomarkers. Limitations include the relatively small sample size of TD model rats, which may impact the representativeness and statistical power of the results. Furthermore, while numerous neurotransmitters exist *in vivo*, this study examined only six selected neurotransmitters, potentially overlooking other underlying mechanisms. Additionally, the scoring of motor and stereotyped behaviors in TD model rats carries inherent subjectivity. Future research could integrate neurotransmitter level changes with techniques such as functional magnetic resonance imaging or electroencephalography to explore more objective biomarkers for diagnosis and efficacy evaluation; metabolomics and proteomics approaches could also be combined to comprehensively and systematically analyze the effects of ARI on neurotransmitters in TD.

## 5 Conclusion

ARI alleviates tic symptoms in TD rats by improving locomotor and stereotyped behaviors, supporting its potential clinical translation for TD treatment. TD rats exhibit dysregulation of Glu and GABA levels, and ARI ameliorates this neurotransmitter dysfunction by modulating these levels. Furthermore, the predictive efficacy of striatal (central) neurotransmitters for TD is significantly superior to that of plasma neurotransmitters, indicating that changes in central neurotransmitters offer better auxiliary diagnostic value for TD.

## Data Availability

The raw data supporting the conclusions of this article will be made available by the authors, without undue reservation.
